# Editorial: The Role of Light in Abiotic Stress Acclimation

**DOI:** 10.3389/fpls.2020.00184

**Published:** 2020-02-25

**Authors:** Tibor Janda, Éva Hideg, Radomíra Vanková

**Affiliations:** ^1^Agricultural Institute, Centre for Agricultural Research, Martonvásár, Hungary; ^2^Department of Plant Biology, University of Pécs, Pécs, Hungary; ^3^Institute of Experimental Botany, Academy of Sciences of the Czech Republic, Prague, Czechia

**Keywords:** abiotic stress, carbon balance, crosstalk, drought, light, osmotic stress, photoinhibition, temperature

Understanding of mechanisms by which plants perceive environmental signals, transmit them to cellular machinery and activate adaptive responses is of fundamental importance from both theoretical and practical points of view. Mutual interactions among environmental factors are inevitable, and light conditions substantially alter the way plants acclimate to changes in other environmental factors. This Research Topic collected eight articles including one minireview and seven original research papers, which demonstrated that the influence of light on achieving high level of stress tolerance could be as important as the genetic background.

Light as a single stress factor may induce photoinhibition. However, over-excitation of Photosystem II has been shown to induce freezing tolerance in cereals. Results of Szalai et al. suggest that light is essential also for the cold acclimation of chilling sensitive maize plants, and thus photoinhibition can be a necessary evil for the initiation of cold tolerance in plants. The avoidance of the damaging effects of high light intensities is definitely required to protect the photosynthetic apparatus. However, for the protection of the whole plant from a subsequent cold shock, it may be necessary to accept the consequences of light-induced stress-related processes to acquire cold-acclimated plants. Microarray based gene expression study demonstrated the existence of complex regulatory mechanisms and interactions between cold and light signaling processes. Differentially expressed genes were mainly involved, among others, in pathways related to chlorophyll biosynthesis, carboxylic acid metabolism, amino acid, porphyrin or glutathione metabolic processes, ribosome biogenesis and translation (Szalai et al.).

Another example of a positive light effect was reported by Lima-Melo et al. who showed that photoinhibition of photosystem I might protect chloroplasts from oxidative damage in Arabidopsis. Light, on the other hand, is capable of exacerbating low temperature stress. Pérez-Llorca et al. demonstrated that changes in leaf angle acted as the first line of light protection in the Mediterranean semi-deciduous shrub *Cistus albidus* during the winter. Furthermore, these plants enhanced accumulation of α-tocopherol, as a stress sensing and signaling mechanism, to counteract combined high light and low temperature stress when modification of the leaf angle was not sufficient defense. Another interesting example of morphological adaptation was described by Shelef et al. who showed that sub-epidermal air spaces in *Silybum marianum* leaves contribute to elevation of leaf temperature; while white leaf patches provide a microenvironment promoting photosynthesis in cold winter morning periods.

Recent results also suggest that not only light intensity but also its quality is important in the regulation of the acclimation processes. The possible involvement of cryptochromes has been reviewed by D’Amico-Damião and Carvalho. Cryptochromes absorb blue and UV-A light, and evoke a wide range of plant stress responses *via* the regulation of several transcription factors, including HY5 (ELONGATED HYPOCOTYL 5) and PIF4 (PHYTOCHROME INTERACTING FACTOR 4). Cryptochrome mediated stress response genes play key roles in signaling pathways, biosynthesis of plant hormones, regulation of reactive oxygen species, or other stress-related components. However, the exact mode of action of cryptochromes or other photoreceptors is still not fully understood. The interactions between the light-related processes and the acclimation mechanisms to osmotic stressors, such as drought and/or high salinity is also very important from both theoretical and practical points of view. Proline is one of the HY5 inducible protective metabolites responding to several factors, especially to osmotic stress. Kovács et al. demonstrated that light-regulated HY5 activates the *P5CS1* (Δ1-PYRROLINE-5-CARBOXYLATE SYNTHASE 1) gene responsible for proline synthesis, suggesting that HY5 promotes proline biosynthesis through connecting light and stress signals. These authors also showed that the activation of the photosynthetic electron transport also promotes proline accumulation in a yet unknown way.

Study of algae or other lower plants may also reveal important mechanisms, which could be utilized for the improvement of stress tolerance of crop plants. Diatoms, a major group of microalgae, are able to adapt to a broad range of environmental conditions. Reorientation of carbon metabolism may occur under unfavorably changing environment, for example at nutrient deficiency. Heydarizadeh et al. showed that even in conditions of carbon deprivation, diatoms may shift their metabolism toward the production of lipids. Light intensity may also affect the carbon flow among different metabolic pathways. Under low light conditions, accumulation of the storage polysaccharide, chrysolaminarin could be observed. Medium light intensity stimulated predominantly lipid synthesis, while high light resulted in an increase in the amount of proteins (Heydarizadeh et al.).

The effects of different light spectra on the photosynthetic parameters, or the UV light induction of certain ROS-scavenging agents have been widely studied. However, the effect of light quality or light intensity on the changes in the root development is much less known and understood. In a complex physiological study, Klem et al. demonstrated the role of light quality in inducing changes in root and leaf morphology, physiology, and metabolite profile. Interestingly, light quality also affected root length: longer roots could be induced when red light was supplemented with far-red radiation (Klem et al.). Root length can be one of the most critical parameters in adaptation processes, especially to drought conditions. Therefore the investigation of the light-dependent responses of root physiology under stress conditions can be interesting and economically important aim of further studies. The effect of light intensity during the cold acclimation period on certain stress-related processes in the roots have also been demonstrated in young maize plants (Szalai et al.).

In conclusion, there is increasing evidence that light is an important regulator of stress acclimation processes. There are still several open questions concerning molecular mechanisms of the light-regulated signaling processes; and further research is required to address the complex nature of the stress signaling networks, as the direct link between the light perception and the development of stress tolerance is still missing.

## Author Contributions

All authors listed, have made substantial, direct, and intellectual contribution to the work, and approved it for publication.

## Conflict of Interest

The authors declare that the research was conducted in the absence of any commercial or financial relationships that could be construed as a potential conflict of interest.

